# Modeling of Charcot-Marie-Tooth disease in zebrafish

**DOI:** 10.3389/fnmol.2025.1641793

**Published:** 2025-08-04

**Authors:** Małgorzata Korzeniowska née Wiweger, Katarzyna Chabros, Weronika Rzepnikowska, Andrzej Kochański, Dagmara Kabzińska

**Affiliations:** ^1^Laboratory of Protein Engineering, Mossakowski Medical Research Institute, Polish Academy of Sciences, Warsaw, Poland; ^2^Department of Neuromuscular Disorders, Mossakowski Medical Research Institute, Polish Academy of Sciences, Warsaw, Poland

**Keywords:** Charcot-Marie-Tooth disease, CMT, neuropathy, animal models, zebrafish

## Abstract

Charcot–Marie–Tooth (CMT) disease is one of the most common inherited neuromuscular disorders, characterized by progressive peripheral nerve degeneration, muscle weakness, and sensory loss. To date, no effective therapy has been developed for CMT. The extreme genetic heterogeneity of CMT, encompassing mutations in more than 50 genes and the involvement of diverse pathological mechanisms, continues to pose significant challenges for disease modeling and therapeutic development. To address these challenges and interrogate specific hypotheses with greater experimental control, researchers have increasingly turned to alternative model organisms that offer genetic tractability and *in vivo* functional readouts. Zebrafish models have been employed to study hallmark features of CMT, including motor deficits, sensory dysfunction, skeletal abnormalities, and auditory neuropathy. Through the use of forward and reverse genetic screening approaches, as well as transgenic lines, zebrafish have yielded some interesting insights into the functional roles of specific genes implicated in CMT and the effects of pathogenic mutations. Moreover, zebrafish serve as a versatile platform for evaluating potential therapeutic interventions, including pharmacological compounds and gene therapy strategies. This review underscores the value of zebrafish as a robust model for advancing our understanding of CMT pathophysiology. It also addresses the ongoing challenges in genetic diagnosis and highlights the therapeutic potential of this model in guiding future treatments for CMT.

## 1 Introduction

Inherited peripheral neuropathies represent a broad, heterogeneous group of genetic disorders. They include hereditary sensory-motor conditions, also known as Charcot-Marie-Tooth diseases (CMT), first described by Charcot (1886), distal hereditary motor neuropathies (dHMN), hereditary sensory autonomic neuropathies (HSAN), and hereditary neuropathy with pressure palsies (HNPP). These conditions share partial phenotypic and genetic overlap. Therefore, for the sake of clarity and consistency, the term “CMT” is used throughout this text to encompass the entire spectrum of hereditary motor, sensory, and sensorimotor neuropathies. CMT diseases are the most common inherited neurological condition, with an estimated global prevalence of 1 in 2,500 individuals, but there is substantial variation in prevalence across different regions (Barreto et al., 2016; Skre, 1974).

Clinical manifestations of CMT are highly variable, but typically include muscle weakness and atrophy, typically beginning in the distal muscles of the feet and hands and progressing proximally. Patients often present with foot drop, steppage gait, and decreased or absent deep tendon reflexes. Sensory deficits, particularly affecting pain and temperature perception, as well as proprioception, are also typical and contribute to gait instability and balance difficulties. In some cases, autonomic symptoms such as orthostatic hypotension, bladder dysfunction, and sweating abnormalities may occur. Skeletal abnormalities, including pes cavus (high-arched feet), hammer toes, hand deformities, and scoliosis, frequently arise due to muscle imbalance and weakness (Cortese et al., 2019; Laurá et al., 2019). In recessive forms of the disease, onset typically occurs during the first decade of life. In contrast, dominant forms most commonly manifest in the third or fourth decade; however, cases with very late onset, even in the seventh decade, have also been reported. The age of symptom onset is also influenced by the specific gene involved, the type of mutation (e.g., missense, deletions, insertions, nonsense mutations), and the location of the mutation within the protein.

Charcot-Marie-Tooth is classified according to inheritance patterns and the predominant type of nerve pathology. The major subtypes include CMT Type 1 (CMT1), CMT Type 2 (CMT2), Intermediate CMT (DI-CMT), CMT Type 4 (CMT4), and X-linked CMT (CMTX). Sensory neuropathies are divided into eight types and sixteen subtypes from HSANI to HSANVIII, while motor neuropathies encompass twenty-four to over thirty types of dHMN depending on the classification adopted (Bird, 1993; Pisciotta and Shy, 2023; Schwartzlow and Kazamel, 2019; Tazir and Nouioua, 2024). CMT1, a demyelinating form, is typically caused by mutations in genes encoding myelin proteins such as *PMP22*, *MPZ*, and *GJB1*. This subtype is characterized by slowed nerve conduction velocities due to myelin sheath abnormalities. CMT2, an axonal form, results from mutations in genes involved in axonal structure and function, such as *MFN2*, *RAB7*, *HSPB1*, and presents with normal or mildly reduced nerve conduction velocities but marked axonal degeneration. Intermediate CMT exhibits characteristics of both demyelination and axonal loss and is often associated with mutations in *DNM2* and *YARS*. CMT4 comprises autosomal recessive forms involving various genes and clinical phenotypes. X-linked CMT, primarily caused by mutations in *GJB1* encoding connexin 32, typically affects males more severely.

CMT diagnosis involves a comprehensive clinical assessment, family history evaluation, electrophysiological studies, and genetic testing. Neurological examination is critical to delineate patterns of weakness, atrophy, and sensory loss. Family history can provide essential clues regarding inheritance. Electrophysiological studies, including nerve conduction velocity and electromyography, help distinguish between demyelinating and axonal forms. Genetic testing using next-generation sequencing panels or whole-exome sequencing confirms the diagnosis and facilitates genetic counseling.

Genetic characterization of hereditary neuropathies began in the late 20th century. However, the term “CMT genes” is variably defined, with classifications encompassing approximately 50 to over 150 genes, often including other syndromes in which neuropathy is a major component of the phenotype. In pure forms of CMT, the number of associated genes is estimated to be between 50 and 60. However, when broader phenotypes are considered, such as genetic syndromes in which neuropathy is part of the clinical presentation, the number of implicated genes increases to approximately 150. This broad inclusion underscores the considerable genetic heterogeneity of the disorder. In classic sensory-motor neuropathy, over 50 genes were described. A small number of mutations, such as those in *PMP22*, *MPZ*, *MFN2*, and *GJB1*, account for over 90% of diagnosed cases (Murphy et al., 2012), while others, like *GDAP1*, are rare and often family-specific (Kabzińska et al., 2022). A common cause of CMT is a 1.4 Mb duplication on chromosome 17 (Lupski et al., 1991; Raeymaekers et al., 1992). Currently, more than 30 genes are associated with motor neuropathies, among them some genes were identified as capable of causing both pure motor neuropathy and classic CMT, such as *HSPB1*, *HSPB8, SORD*, and *DNAJB2* (Tazir and Nouioua, 2024). Similarly, 15 genes of sensory neuropathy have been described, like *SPTLC1*, *ATL1*, *NTRK1*, and *SCN9A* (Schwartzlow and Kazamel, 2019). The number of identifiable genes has progressively increased with advancements in next-generation sequencing (NGS)-based diagnostic technologies. Non-Mendelian inheritance patterns, including multilocus and oligogenic inheritance, have also been proposed (Bis-Brewer et al., 2020), and some mutations can exhibit both dominant and recessive inheritance (Rzepnikowska and Kochański, 2018). The molecular diagnosis is further complicated by weak-effect sequence variants, structural mutations (Cutrupi et al., 2018; Gonzaga-Jauregui et al., 2015), and the ambiguous pathogenicity of specific genetic alterations.

Variants are classified into five categories: benign, likely benign, variant of uncertain significance (VUS), likely pathogenic, and pathogenic based on ACMG guidelines (Richards et al., 2015). VUS remain particularly problematic in poorly characterized genes such as *WARS1*, *SARS1*, and *RAB40B* (Favalli et al., 2021). Conflicting variant interpretations further complicate diagnostics; for example, *GJB1* shows a 7.3% conflict rate. In *GARS1*, 49% of variants are VUS and only 8% are classified as pathogenic. Similarly, *DNM2* mutations, linked to both myopathy and intermediate CMT, include 43.5% VUS and just 4.6% pathogenic variants (Koutsopoulos et al., 2011). For *MFN2*, implicated in CMT2A, less than 20% of variants are pathogenic, with over 50% remaining as VUS (Beręsewicz et al., 2018; Züchner et al., 2006). The inconsistency of bioinformatics tools used for pathogenicity prediction underscores the urgent need for improved variant interpretation methods. Most CMT-associated variants have not been functionally validated, as such analyses often lie outside the scope of routine diagnostics. Despite technological advances, only about 50% of CMT cases are genetically diagnosed (Drew et al., 2015; Schabhüttl et al., 2014), with even lower diagnostic yields in HMN and HSAN subgroups (Cortese et al., 2019).

Currently, there is no cure for CMT, and applied therapies focus on symptomatic treatment, maintaining mobility, and improving quality of life. Physical and occupational therapy, alongside assistive devices and customized exercise programs, can help preserve muscle function. Orthopedic interventions, including surgical correction of deformities and orthotic support, aid mobility and pain management. Medications such as gabapentin, pregabalin, and NSAIDs are used to treat neuropathic pain. Genetic counseling provides essential guidance on inheritance, recurrence risks, and reproductive options.

Ongoing advancements in molecular biology and genetics offer hope for targeted therapies.

## 2 New therapeutical approaches for CMT diseases

Numerous novel therapeutic strategies have been proposed (Okamoto and Takashima, 2023; Pisciotta et al., 2021; Stavrou et al., 2021), offering hope for the development of effective treatments. Several compounds have undergone clinical testing. Among those demonstrating acceptable safety profiles but limited or inconclusive efficacy are PXT3003 and epalrestat. PXT3003 is being developed for the treatment of CMT1A, the most prevalent CMT subtype, caused by a *PMP22* gene duplication. It is a combination of baclofen, naltrexone, and sorbitol, three drugs approved for other indications, formulated as an oral solution. In preclinical studies, PXT3003 modestly reduced *PMP22* expression, enhanced myelination, increased the number and normalized the size of functional neuromuscular junctions (NMJs), and generally improved the clinical phenotype in CMT1A transgenic rat models (Chumakov et al., 2014; Prukop et al., 2020). A Phase II clinical trial (NCT01401257) provided preliminary evidence of PXT3003’s efficacy and safety in CMT1A patients (Attarian et al., 2012). In the Phase III trial (NCT02579759), the high-dose group demonstrated statistically significant improvement in the primary endpoint. However, concerns regarding the stability of the high-concentration formulation emerged (Attarian et al., 2021), prompting the initiation of a new clinical trial in 2021 (NCT04762758).

Applied Therapeutics has developed a next-generation aldose reductase inhibitor (ARI), AT-007 (govorestat), which effectively inhibits the conversion of glucose to sorbitol. Preliminary results from the INSPIRE clinical trial (NCT05397665) in Sorbitol Dehydrogenase (SORD) Deficiency using AT-007 demonstrated a significant reduction in sorbitol levels in patients (averaging 52%) compared to the placebo group and a statistically significant correlation between sorbitol level, the pre-specified CMT-FOM composite clinical endpoint, and the CMT Health Index (De Grado et al., 2025; GlobeNewswire, 2024; Zhu et al., 2023). *SORD* encodes sorbitol dehydrogenase, the second enzyme in the polyol pathway, where glucose is first converted into sorbitol by aldose reductase and then into fructose by SORD. Loss-of-function mutations in *SORD* lead to sorbitol accumulation in cells and plasma (Cortese et al., 2020). Another drug, epalrestat, an aldose reductase inhibitor, blocks the conversion of glucose to sorbitol and has significantly reduced sorbitol levels in fibroblasts derived from SORD-CMT patients (Cortese et al., 2020). Epalrestat is indicated primarily for the management of diabetes-related complications, particularly diabetic peripheral neuropathy. While it does not exert direct neurodegenerative effects, its ability to mitigate hyperglycemia-induced neuronal injury allows for indirect neuroprotection and preservation of peripheral nerve function. The therapeutic effect of epalrestat is based on the inhibition of aldose reductase. Under hyperglycemic conditions, excessive intracellular accumulation of toxic sorbitol in neuronal tissue contributes to osmotic stress, oxidative damage, and subsequent cellular dysfunction (Li et al., 2016; Ran et al., 2024). A similar effect has been observed in animal models as well as in patients with CMT caused by mutations in the *SORD* gene. By reducing sorbitol levels, epalrestat may attenuate or delay the progression of neuropathy and associated nerve cell damage (Prukop et al., 2020). It is currently approved in several countries for treating diabetic complications and has demonstrated a favorable safety profile (Grewal et al., 2016). A clinical trial evaluating epalrestat’s safety and efficacy for SORD CMT2 was registered in 2023, although recruitment has not yet commenced (NCT05777226).

Gene therapy is among the most actively pursued therapeutic approaches for genetic disorders, including CMT. It encompasses techniques aimed at suppressing disease phenotypes by replacing, modifying, silencing, or repairing defective genetic material in patient cells. Tailored strategies may be required depending on the underlying genetic mechanism. For loss-of-function mutations, gene replacement is typically indicated, whereas dominant-negative or toxic gain-of-function mutations may benefit from gene silencing, editing, or dosage reduction (Stavrou et al., 2023). The majority of gene therapies for CMT are still in the preclinical stage of development (Stavrou et al., 2023). One therapy that has advanced further is VM202, a non-viral, intramuscularly delivered synthetic cDNA hybrid encoding human hepatocyte growth factor (HPHGF). This therapy aims to stimulate nerve regeneration (Ko et al., 2018). A Phase I/IIa clinical trial (NCT05361031) evaluated its safety and tolerability of in patients with CMT1A caused by *PMP22* duplication.

A separate investigational approach involves neurotrophin-3 (NT-3), a neurotrophic factor essential for Schwann cell survival and nerve regeneration (Sahenk and Ozes, 2020). Although a Phase I/IIa trial was initiated for CMT1A patients, it is currently suspended due to vector production issues (NCT03520751). In parallel, another early-stage clinical trial is underway to deliver a functional *IGHMBP2* gene for treating IGHMBP2-related neuropathies, including CMT2S (NCT05152823).

Another promising avenue involves the use of stem cell-based therapies. Mesenchymal stem cells (MSCs) offer neuroprotective effects and promote regeneration by secreting antioxidant, antiapoptotic, and immunomodulatory molecules. They have shown efficacy in remyelination processes (Yousefi et al., 2019). A completed Phase I study (NCT05333406) assessed the safety and dosing of a single intravenous administration of allogeneic umbilical cord-derived MSCs (EN001) in nine CMT1A patients, with no serious adverse reactions reported. As a follow-up, a clinical trial was registered for CMT1E (caused by point mutations in *PMP22*) (NCT06218134).

Currently, recruitment is ongoing for a Phase I trial of CLZ-2002 in CMT1 patients. This trial will evaluate the safety and tolerability of intramuscular injections of allogeneic MSC-derived neuronal regeneration-promoting cells (Schwann cell-like cells) (NCT05947578).

## 3 Advantages and limitations of models used in CMT research

Animal and cellular models have provided crucial insights into human disease mechanisms and therapeutic development, including for genetic disorders such as CMT. Numerous rodent models of CMT have been successfully developed and extensively characterized (Bosco et al., 2021; Juneja et al., 2019). An additional valuable mammalian model includes dogs, in which spontaneous mutations have led to naturally occurring inherited neuropathies that resemble human CMT. Such neuropathies have been identified in at least 22 dog breeds (Granger, 2011). Dogs offer several advantages as disease models, including larger body size, longer lifespan, and greater physiological similarity to humans compared to rodents (Drögemüller et al., 2010). Moreover, as companion animals, they share environmental exposures with humans, adding ecological relevance to disease studies (Skedsmo et al., 2019). Despite these benefits, mammalian models are typically expensive and time-consuming to maintain, and their use raises ethical concerns. Therefore, alternative systems for CMT modeling that adhere to the 3Rs: principle Replacement (whenever possible to use other methods and models to replace the mammals), Reduction (to use the minimal number of animals that is needed to obtain statistically valid results), and Refinement (to minimize animal’s burden during experiment) should be employed whenever feasible.

Beyond animal models, several cellular systems have been established to study CMT pathogenesis. Although yeast models have significant limitations, including a lack of neuronal complexity, absence of genes involved in myelination, and inability to simulate interactions between different cell types, they remain useful for investigating basic cellular mechanisms, screening potential therapeutic compounds (Binięda et al., 2021; Qiu et al., 2023), and identifying candidate targets for intervention (Rzepnikowska et al., 2020a; Rzepnikowska et al., 2020b; Rzepnikowska et al., 2022). Organoids derived from human induced pluripotent stem cells (iPSCs) offer another advanced model system, capable of mimicking complex cellular environments. CMT1A-specific iPSC-derived organoids containing neurons, Schwann cells, muscle cells, endothelial, and glial cells have been developed (Van Lent et al., 2022). These models enable the study of axonal myelination and intercellular interactions. However, a significant limitation is the absence of directional cell growth, which contrasts with the *in vivo* development of the peripheral nervous system. Consequently, organoids may not be suitable for neuromuscular junction (NMJ)-focused studies (Van Lent et al., 2022). iPSCs are widely employed in disease modeling due to their human origin, high differentiation potential, and accessibility from skin fibroblasts or blood cells. Both patient-derived and genetically engineered iPSC-derived motor neurons serve as relevant tissue models for investigating disease mechanisms and identifying candidate therapies (Feliciano et al., 2021; Perez-Siles et al., 2020; Saporta et al., 2015; Van Lent et al., 2021). Nevertheless, traditional 2D and 3D cultures cannot replicate the full cellular complexity of the peripheral nervous system, limiting their utility, particularly for modeling demyelinating CMT types. While Schwann cells have been generated from human iPSCs (Liu et al., 2012), they - like primary human Schwann cells - have failed to robustly myelinate iPSC-derived neurons *in vitro*. Notably, myelination has been observed in co-cultures involving iPSC-derived neurons and rat-derived myelinating Schwann cells (Clark et al., 2017).

More complex yet scalable models include the nematode *Caenorhabditis elegans* and the fruit fly *Drosophila melanogaster*, both of which are advantageous for high-throughput screening and functional genetic studies. These invertebrates have been used to assess behavioral, cellular, and molecular effects of CMT-related mutations (Cortese et al., 2020; El Fissi et al., 2018; Kitani-Morii and Noto, 2020; Lin et al., 2022; López Del Amo et al., 2015; Brozkova et al., 2015; Soh et al., 2020). However, a significant limitation of these organisms is the absence of Schwann cells and myelinated axons, making them unsuitable for modeling demyelinating forms of CMT (Chung et al., 2020). In contrast, fish models such as zebrafish overcome all these limitations.

## 4 The zebrafish model of CMT

**FIGURE 1:**
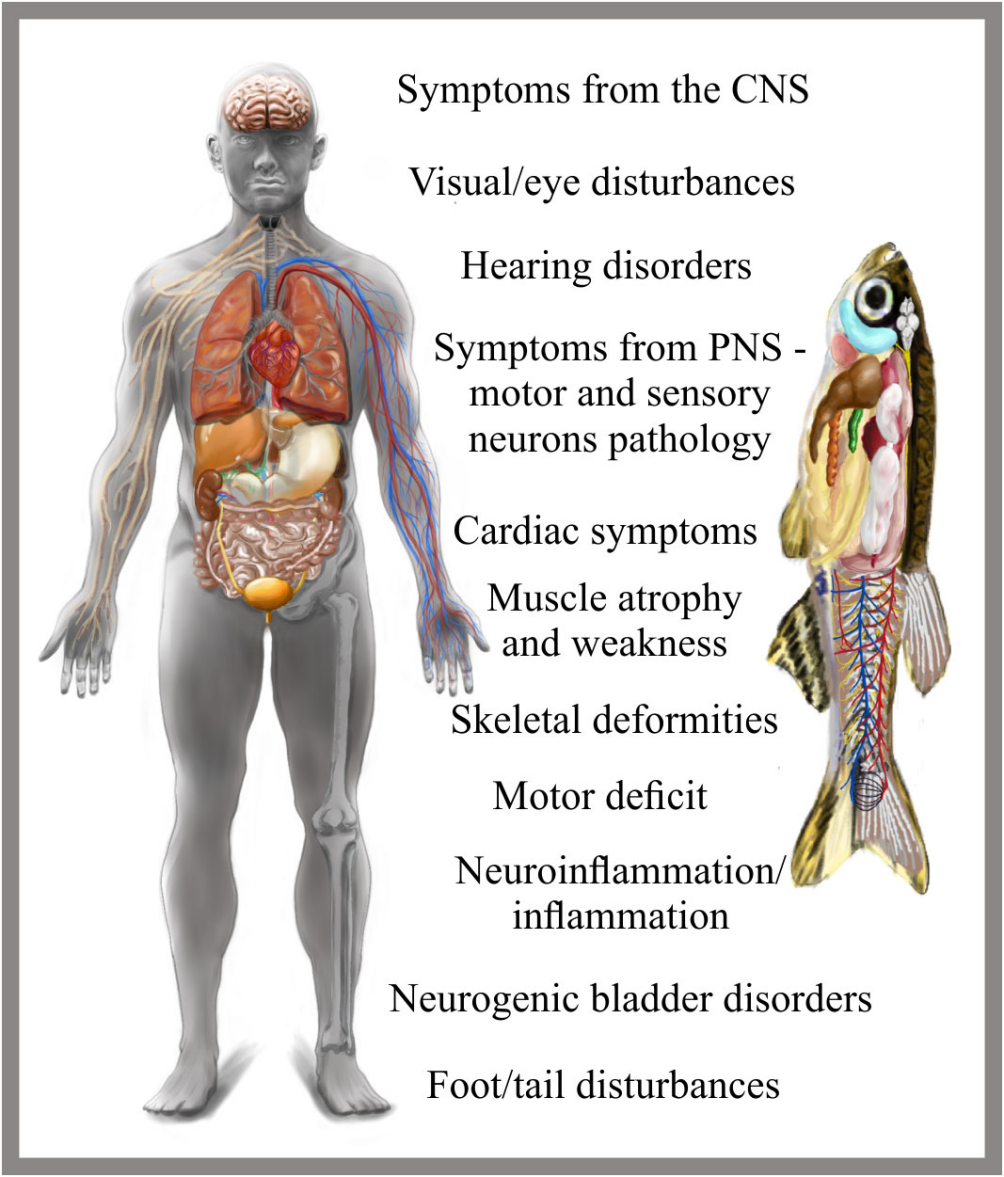
The usefulness of zebrafish in modeling hereditary neuropathies. Zebrafish serve as a valuable model organism for studying a wide range of human disorders, including hereditary neuropathies. The illustration highlights various human physiological systems and the corresponding CMT disease symptoms that can be effectively modeled using zebrafish.

An ideal model organism for studying neuropathies should have a well-characterized and accessible nervous system, a conserved neuromuscular architecture, and the ability to replicate key aspects of human pathology, including axonal degeneration, demyelination, and neuromuscular dysfunction. Despite notable differences in structure, complexity, and remarkable regenerative capacity, zebrafish fulfill these criteria (Figure 1). It shares significant anatomical and functional similarities with humans in their neuromuscular systems. Both species have a central nervous system (CNS) comprising the brain and spinal cord, and a peripheral nervous system (PNS) consisting of sensory and motor neurons responsible for regulating crucial processes, such as the strength of muscle contractions, which are impaired in CMT (Babin et al., 2014; Singh and Patten, 2022). Although the zebrafish PNS has fewer types of sensory neurons and a less complex branching pattern in the peripheral nerves compared to humans or other mammalian models of CMT, it performs similar functions. At early stages of development, the zebrafish PNS is highly accessible for live imaging, making it a valuable research tool (Chia et al., 2022; Xiao et al., 2015). In zebrafish, peripheral axons are myelinated, though the myelin sheets are thinner and begin forming only after functional axons are established, typically starting at 3–5 days post fertilization (dpf) (D’Rozario et al., 2017). Similarly, as in humans, zebrafish myotomes derived from somites contain three distinct types of muscle fibers (slow, fast, and intermediate). These fibers are organized into repeating units called myomeres, which are divided by a connective tissue (myoseptum) into structural and functional units. However, unlike mammals, zebrafish slow and fast muscles are spatially segregated - slow muscle fibers are located on the superficial (outer) layer of the myotome, while fast muscle fibers occupy the deeper (inner) layers (Daya et al., 2020). This spatial organization provides a unique opportunity to investigate how specific motor neurons target different muscle fiber types, how these connections are affected by neuromuscular disorders like CMT, and how fiber-typespecific deficits contribute to motor dysfunction. Additionally, this segregation simplifies the assessment of fiber-type-specific regeneration or degeneration in response to nerve or muscle damage, enhancing the zebrafish’s utility as a model organism for neuromuscular research.

In addition to their utility in studying neuromuscular connections, zebrafish also provide valuable insights into secondary complications associated with CMT, including skeletal abnormalities. Due to the aquatic environment and the buoyancy it provides, the zebrafish skeleton is not subjected to the same gravitational loading experienced by humans. Nevertheless, zebrafish can develop different axial deformities, including age- or disease-related spine deformities and idiopathic scoliosis (Boswell and Ciruna, 2017). Furthermore, zebrafish can be used to study defects in bone mineralization, vertebral segmentation, or skeletal growth, providing insights into the genetic and molecular mechanisms underlying these conditions (Carnovali et al., 2019; Marí-Beffa et al., 2021; Van Hul et al., 2020).

Hearing deficits in CMT, often linked to auditory neuropathy, can also be effectively modeled in zebrafish (Bever and Fekete, 2002; Vona et al., 2020). Although zebrafish lack a cochlea, which limits their ability to replicate the complex auditory processes seen in humans, their inner ear and lateral line system share structural and functional similarities with mammalian auditory systems, including conserved stereocilia architecture, synaptic mechanisms, and neuronal connectivity (Kindt and Sheets, 2018; Whitfield, 2002). Moreover, the lateral line system is externally accessible and exhibits robust hair cell regeneration, providing a unique platform for studying mechanisms of auditory damage and repair (Hardy et al., 2021). Ototoxic stress can be induced using drugs or environmental stimuli (Domarecka et al., 2020), enabling studies of the cellular and molecular responses to such stressors, facilitating the identification and evaluation of potential therapeutic targets.

The cardiac system in zebrafish also offers valuable insights into CMT-related complications, such as arrhythmias and conduction disturbances in association with peripheral muscle atrophy. Although zebrafish hearts have only a single atrium and ventricle, they share key physiological properties, including similar heart rate and action potential duration, conserved ion channels, conduction pathways, and autonomic regulation of heart function (Tesoriero et al., 2023). Zebrafish are particularly well-suited for real-time imaging of cardiac activity, making them a powerful tool for studying heart function. Additionally, zebrafish models enable the investigation of the role of the autonomic nervous system in regulating heart rate and rhythm, which is often disrupted in CMT (Pedroni et al., 2024).

Some authors suggest that neurogenic bladder disorders that result from peripheral neuropathy, which disrupts the normal communication between the bladder and the nervous system, are associated with CMT. The presence of the urinary bladder has been confirmed in some teleost fish, though its existence in zebrafish was previously questioned. Recent findings by the Catto group demonstrated that the zebrafish urinary bladder is present in adult zebrafish (Jubber et al., 2023) but in contrast to the multi-layered human urothelium, zebrafish urinary bladder is lined by epithelium composed of one or two cell layers, expressing proteins characteristic of both superficial (uroplakins) and basal (Cytokeratin 5 and CD44) layers of human urothelium. Using fluorescent dye, Jubber et al. (2023) showed that the urine accumulates in the zebrafish urinary bladder and is intermittently released via a distinct urethra. While the responses of the urinary bladder to various stimuli have been described in the Atlantic cod (Nilsson, 1970), similar studies in zebrafish are still lacking.

Sweating abnormalities can significantly impact the quality of life in CMT patients. Although fish lack sweat glands and are therefore not suitable for studying sweating dysfunctions in the traditional sense, zebrafish provide a valuable model for assessing autonomic dysfunctions, such as impairments in temperature regulation and sympathetic nervous system function. For example, zebrafish can be tracked as they navigate through a thermal gradient to select their preferred environmental temperature, thereby achieving temperature homeostasis (López-Olmeda and Sánchez-Vázquez, 2011; Palieri et al., 2024). In this way, zebrafish offer key insights into thermal regulation and its impact on broader physiological processes. However, these studies have not yet been conducted in the context of CMT.

To elucidate the molecular and cellular mechanisms underlying CMT in zebrafish and explore potential therapeutic strategies, a variety of experimental approaches can be employed. For example, mitochondrial function, axonal transport, and myelination can be chemically modulated (Azevedo et al., 2020; Toni et al., 2023). In most cases, substances are directly added to the fish water, making this type of experiment straightforward and highly efficient in terms of time, cost, and labor. This approach is particularly advantageous for zebrafish embryos and larvae, which are typically maintained in Petri dishes or multi-well plates, containing relatively small volumes of liquid, thereby enabling effective use of limited quantities of test substances. The availability of diverse transgenic zebrafish lines further amplifies the utility of the zebrafish model by enabling the visualization of cellular events at high resolution. For example, *Tg(hb9:MTS-Kaede)* line was used to visualize mitochondrial dynamics in motor neurons and the effects of CMT2A-causing mutations on mitochondrial movement (Bergamin et al., 2016), and with *Tg(TagRFP-caax)*, it was possible to assess the effects of CMT2b-associated alterations on long projection sensory neurons (Ponomareva et al., 2016). Transgenic lines, like *Tg(mbp:nfsB-egfp)* in which bacterial nitroreductase enzyme (NTR) converting metronidazole into a cytotoxic compound is driven under oligodendrocyte-specific promoter, can be used, for e.g., selective and reversible ablation of oligodendrocytes and subsequent demyelination upon treatment with metronidazole (Chung et al., 2013). As zebrafish have an amazing regeneration capacity, once metronidazole is withdrawn, this transgenic system offers the possibility to study remyelination.

Reverse genetic screens were also effective and facilitated efficient and rapid investigation across various genetic backgrounds, allowing for the precise identification of the roles of different genes and modifiers. Among the methods used to study gene function in model organisms, siRNA-mediated knockdown is generally not effective in zebrafish. In contrast, morpholino oligomers (MOs), which typically are ∼ 25-nucleotide molecules designed to block translation or alter splicing by binding to target mRNAs/pre-mRNAs can be used to create morphants – zebrafish embryos and larvae with robust but transient gene knockdown (Stainier et al., 2017; Vettori et al., 2011). Although the use of MOs can be advantageous when studying early development in hypomorphic conditions, however, in other cases, the incomplete knockdown and off-target effects findings should be validated with methods complementary to MO. Since the CRISPR/Cas9 technology has revolutionized genome editing, both transient genetic modifications (crispants) and stable edits via non-homologous end joining (NHEJ) can be created with high efficiency, whereas homology-directed repair (HDR), a key genome editing mechanism in mammalian models like mice, remains far less efficient in zebrafish compared to NHEJ. In addition to morphants and crispants, dominant-negative effects of different genes or their modulators can also be assessed by injecting DNA, RNA, or proteins into one-cell zebrafish embryos and observing their impact on developing embryos (Hong et al., 2016; Mullen et al., 2021).

Dozens of CMT and neuropathy-related genes have been studied in zebrafish (Table 1), one of which is the *RAB7* gene. The zebrafish Rab7a shares 97.6% amino acid identity with the human RAB7 protein, with 100% identity at the residues affected in the human disease, specifically L129F, K157N, N161T, and V162M. To study the role of *rab7a* in the axon growth and guidance defects during sensory neuron development Ponomareva and colleagues (Ponomareva et al., 2016) created constructs in which mutated *rab7a* was placed under control of cis-regulatory elements from the *neurogenin 1* gene, driving expression to Rohon-Beard (RB) spinal sensory neurons. Transient expression was obtained by injecting constructs into one-cell stage embryos, allowing the first analysis already at 23 hours post fertilization (hpf), when the RB neurons start to develop. Using the same constructs and Tol2 transposase stable transgenic lines: *Tg(-3.1ngn1:GFP-Rab7)*, *Tg(-3.1ngn1:GFP-Rab7L129F)*, and *Tg(-3.1ngn1:GFP-Rab7K157N)*, with CMT2b *Rab7* mutations in spinal sensory neurons only were generated (Ponomareva et al., 2016). Using those tools, the authors demonstrated that, as in patients, mutations in *rab7a* caused neurodevelopmental defects. Moreover, reduced axon growth and branching most likely resulted from the expression of a constitutively active form of Rab7a. Tol2 is still used as an efficient tool for random integration of larger DNA fragments into the zebrafish genome, and humanized zebrafish transgenic lines like the *Tg (DNM2WT-EGFP)*, which was created to study subcellular localization of DNM2-EGFP in skeletal muscle cells, is an example of this application (Zhao et al., 2019).

**TABLE 1:** Charcot–Marie–Tooth-related genes investigated in zebrafish.

Gene	Zebrafish orthologs	Disease phenotypes	Zebrafish phenotype	References
*AARS1*	*aars1*	CMT2N	Reduction in axon length	Jin et al., 2022; Weterman et al., 2018
*ABHD12*	*abhd12*	PHARC	Aberrant axon extension, branching	Gonzaga-Jauregui et al., 2015
*ATL1*	*atl1*	HSNID	Abnormal architecture of spinal motor axons	Fassier et al., 2010
*CHCHD10*	*chchd10*	dHMN-VIIB	Motoneuron pathology, abnormal myofibrillar structure, and mobility deficits	Brockmann et al., 2018; Petel Légaré et al., 2023
*DCTN1*	*dctn1a dctn1b*	dHMN7B	Defects in the development of spinal cord motor neurons and the function of the neuromuscular junction	Bercier et al., 2019
*DGAT2*	*dgat2*	CMT2	Inhibited axonal branching	Hong et al., 2016
*DNM2*	*dnm2a* *dnm2b*	CMT2M DI-CMT B CNM1 MSL	Defects in muscle morphology, defects in motor neuron formation, with incorrect branching or total absence of branching	Bragato et al., 2016; Gibbs et al., 2013; Gibbs et al., 2014
*FBLN5*	*fbln5*	CMT1H HNARMD	Myelination defects	Won et al., 2020
*FIG4*	*fig4a fig4b*	CMT4J	Robust liver vacuolation	Bao et al., 2021
*GARS1*	*gars1*	CMT2D HMND5	Pericardial edema Developmental defects (unconsumed yolk and minor head and body axis)	Malissovas et al., 2016
*GBF1*	*gbf1*	CMT2GG	Vascular collapse and hemorrhage	Chen et al., 2017; Dutton et al., 2009
*GDAP1*	*gdap1*	CMT2K CMT2H RI-CMTA CMT4A	Reduced density of sensory neurites, decreased temperature–related activity	Gonzaga-Jauregui et al., 2015
*HARS1*	*hars*	CMT2W	Reduction in axon length	Mullen et al., 2021; Waldron et al., 2017
*HOXD10*	*hoxd10a*	CMT1	Locomotor behavior, vertebral identity, and peripheral nervous system development alteration	de la Cruz et al., 1999; Shrimpton et al., 2004
*HNRNPA1*	*hnrnpa1a hnrnpa1b*	HMN	edema, abnormal intersegmental vessels branching	Liu et al., 2017
*HSPB1*	*hspb1*	CMT2F dHMN2B	Reduction in the cross-sectional area of myofibers	Gonzaga-Jauregui et al., 2015; Middleton and Shelden, 2013
*HSPB8*	*hspb8*	CMT2L dHMN2A	Overall reduction of the birefringence of muscles Decreased locomotor activity	Dubińska-Magiera et al., 2020; Mao et al., 2005
*KARS1*	*kars1*	RI-CMTB	Morphological abnormalities (heart edema, smaller heads, eyes, otic vesicle) Abnormal trunk muscle fibers failed to inflate the swim bladder Failed to respond to touch and displayed a loss of spatial orientation Reduced eye and head axial length Loss of locomotor activity in response to light or acoustic startle	Lin et al., 2021
*KIF1A*	*kif1aa kif1ab*	HSN2C	Extensive locomotor activity	Guo et al., 2020
*KIF1B*	*kif1b*	CMT2A1	Disturbances of myelination in the nervous system and outgrowth of some of the longest axons in the peripheral and central nervous systems	Lyons et al., 2009
*LAS1L*	*las1l*	SMARD2	Early lethality and disruption of muscle and peripheral nerve architecture	Butterfield et al., 2014
*LITAF*	*litaf*	CMT1C	Promotes inflammatory responses and activates apoptosis	Chen et al., 2021
*LRSAM1*	*lrsam1*	CMT2G CMT2P	Variation in the severity of the phenotype (phenotype varied from near normal with a slightly smaller head, a slightly shorter body axis, slightly less pigmentation and bent tail tips to completely curled up and smaller embryos with bent tails with little pigmentation, smaller eyes, abnormal brain development and a less organized structure of the sometimes) Abnormal swimming behavior	Weterman et al., 2012
*MED25*	*med25*	CMT2B	Axonal defects	Gonzaga-Jauregui et al., 2015
*MFN2*	*mfn2*	CMT2A2A CMT2A2B HMSN6A MSL	Facial prognathism, underdeveloped eyes, brain ventricles enlargement, curly-tail, motor impairment, or completely unresponsive to touch	Gonzaga-Jauregui et al., 2015; Vettori et al., 2011; Zhou et al., 2020
*MPZ*	*mpz*	DI-CMTD CMT1B CMT2I CMT2J DSS CHN2 Roussy- Levy syndrome	Decreased total amount of synthesized myelin membrane and number of myelinated axons	Antonellis et al., 2010; Preston et al., 2019
*NEFL*	*nefla neflb*	DI-CMTG CMT1F CMT2E	Decreased locomotor activity	Demy et al., 2020
*NRG1*	*nrg1*	CMT-DI	Decreased locomotor activity	Lysko et al., 2022; Schonkeren et al., 2019
*PMP22*	*pmp22a pmp22b*	CMT1A CMT1E DSS HNPP Roussy- Levy syndrome	Reduced nerve conduction velocity	Jones et al., 2012
*PMP2*	*fabp4b*	CMT1G	Axonal defects	Gonzaga-Jauregui et al., 2015
*PRPS1*	*prps1a prps1b*	CMTX5	Smaller eyes and reduced hair cell numbers Abnormal development of primary motor neurons, hair cell innervation, and reduced leukocytes	Pei et al., 2016
*RAB40B*	*rab40b rab40c*	CMT2	Defective swimming pattern of stalling with restricted localization and slower mobility	Son et al., 2023
*RAB7A*	*rab7a*	CMT2B	Defects in sensory axon growth, branching, and path finding	Ponomareva et al., 2016
*REEP1*	*reep1*	HMND12 HMNR6	Defects in motor axon outgrowth leading to motor impairment, mitochondrial dysfunction, and reactive oxygen species accumulation	Naef et al., 2023
*SARS1*	*sars1*	NEDMAS	Smaller head and eyes Heart edema	(Bögershausen et al., 2022)
*SBF1 (MTMR5)*	*sbf1*	CMT4B3	Morphometric changes in head size and brain volume, reduced overall body size, complex set of defects in the trunk of the embryo	Lindzon et al., 2025; Ho and Kane, 1990
*SCN9A*	*scn1lab*	Erythermalgi a, primary Insensitivity to pain, congenital HSAN2D PEPD SNFP	Decreased density of the small-nerve fibers Increase in activity induced by temperature change	Eijkenboom et al., 2019a
*SLC25A1*	*slc25a1a*	MCVD	Altered tail morphology Impairment of the escape response induced by touch Abnormal neuromuscular junction development, edema of the hindbrain, heart, yolk sac, and tail	Chaouch et al., 2014
*SOX10*	*sox10*	PCWH	Neurogenesis alterations of olfactory sensory neurons	Saxena et al., 2013
*SPTLC1*	*sptlc1*	HNA1A	Randomized epiblast cell divisions	Castanon et al., 2020
*TFG*	*tfg*	CMT2	Decreased locomotor activity	Chen et al., 2022
*VABP*	*kcnip1a kcnip1b*	SMAFK	Cardiac bradycardias	Silbernagel et al., 2018
*VRK1*	*vrk1*	dHMN dHMN and pyramidal features	Microcephaly and impaired motor function, Decreased cell proliferation, Defects in nuclear envelope formation and heterochromatin formation in the brain	Carrasco Apolinario et al., 2023
*VWA1*	*vwa1*	HMNMYO	Jaw joint, ventral cartilage, arches, Meckel’s and palatoquadrate abnormalities, locomotor behavior disturbances	Pagnamenta et al., 2021
*WARS1*	*wars1*	dHMN9	Smaller head and eyes Heart edema	Bögershausen et al., 2022
*WNK1*	*wnk1a wnk1b*	HSAN2A	Improper peripheral lateral line development	Bercier et al., 2013; Gonzaga-Jauregui et al., 2015

Compared to other vertebrate models, zebrafish, with its easily available large clutches of embryos, offer unique advantages for cost-effective forward genetic screens that allow identification of new genes involved in certain processes. For example, the Tablot group conducted a genetic screen to identify genes that are critical for the development of myelinated axons in zebrafish (Pogoda et al., 2006). In their study, the authors utilized homozygous mutants from the F3 generation, generated through premeiotic mutagenesis with the chemical mutagen ENU (N-ethyl-N-nitrosourea). Their approach involved analyzing the expression of *myelin basic protein* (*mbp*) – a robust marker of myelinating glia in the CNS and PNS. By screening 1859 clutches of F3 larvae from 504 F2 families, they identified 13 mutations affecting 10 genes that are essential for myelinated axon development. Of these mutations, *st23* mapping in the linkage group 23 was pointed out as a novel gene which is likely to be a good model of CMT2 axonal peripheral neuropathies. Later, the Talbot group showed that *st43* mutation affects *kinesin motor protein* (*kif1b)*, a gene which required to localize myelin mRNA to oligodendrocyte processes, ensuring proper myelin sheath formation around axons, and preventing the ectopic production of myelin-like membrane (Lyons et al., 2009). Although this study did not ultimately identify a new gene, the identification of *kif1b* in a forward genetic screen demonstrated the model’s relevance for this type of studies. Another member of kinesin proteins – *KIF5A*, which has two semi-orthologs in zebrafish - *kif5Aa* and *kif5Ab*, also sheds light on Kinesin complexity in CMT and reveals determinants of specific Kif5A functions in mitochondrial transport, adaptor binding, and axonal maintenance (Campbell et al., 2014). Similar to SPG10 patients, zebrafish kif5Aa^*sa*7168^ mutant display striking motor dysfunction. Campbell and co-authors showed that the peripheral sensory axons from the *kif5Aa* mutant lack mitochondria and degenerate. Moreover, concurrent loss of the *kinesin-3*, *kif1b*, or its adaptor *kbp*, exacerbates axonal degeneration via a non-mitochondrial cargo common to Kif5Aa (Campbell and Marlow, 2013). The example also shows that gene duplication, which in CMT related genes is twice higher than the average for the genome (Kozol et al., 2016), does not discredit the usefulness of the model. Instead, it underscores the model’s capacity to account for genetic variations and complexities, which can be essential for understanding and addressing CMT.

The zebrafish model not only enables the exploration of the functions of genes already associated with CMT but also serves as a crucial tool for investigating the effects of new variants. For example, a zebrafish mutant carrying a rare missense variant in *neuregulin 1* (*nrg1*), provided initial evidences supporting the pathogenicity of a homozygous *NRG1* variant identified in a patient with sensory and motor deficits consistent with mixed axonal and demyelinating peripheral neuropathy may cause peripheral neuropathy. These findings suggest that *NRG1* should be further investigated in families with peripheral neuropathy of unknown cause (Lysko et al., 2022). The absence of the desired mutation in the zebrafish genome is not a limiting factor. Three CMT-associated substitutions (V155G, Y330C, R137Q) in the cytoplasmic *histidyl-tRNA synthetase (hars1)* on neurite outgrowth and peripheral nervous system development were also studied in the zebrafish model by injecting Y330C and V155G variants of human *HARS1* mRNA (Mullen et al., 2021). Hong et al. (2016) using similar approach, showed that Y223H *DGAT2* induced an axonal defect in the peripheral nervous system of zebrafish and Talbot group after identifying a rare R > Q missense variant in *NRG1* used zebrafish model to provide evidence indicating that partial loss of NRG1 function indeed may cause peripheral neuropathy in humans (Lysko et al., 2022). By modeling variants of unknown significance, researchers can determine their functional impact, offering valuable information for both clinical interpretation and therapeutic development.

Motor behavior, muscle morphology, and motor neuron in fish over-expressing the G537C mutation in the PH domain of human DYNAMIN-2 were also reflected in human CMT (Bragato et al., 2016). Notably, zebrafish can be used to uncover even more complex scenarios. Holloway and coauthors reported a story of a child with leukemia and no family history of neuropathy who developed severe chemotherapy-induced peripheral neuropathy after vincristine treatment (Holloway et al., 2016). The child was found to have a novel loss-of-function mutation in *GARS*, suspected of predisposing a patient to severe CIPN. The authors successfully modeled the impact of the mutation in morphant and mRNA-injected zebrafish and obtained a similar phenotype as in the patient, both prior to and after the chemotherapy. Moreover, some of the vincristine-induced neurotoxicity and axonal defects were elevated when fish were co-administered with microtubule stabilizing drug paclitaxel (vincristine is a microtubule-destabilizing drug (Holloway et al., 2016). These findings highlight the potential of zebrafish models for studying disease mechanisms and identifying therapeutic strategies, emphasizing the value of drug combination approaches in mitigating chemotherapy-induced side effects.

## 5 Conclusion

**FIGURE 2:**
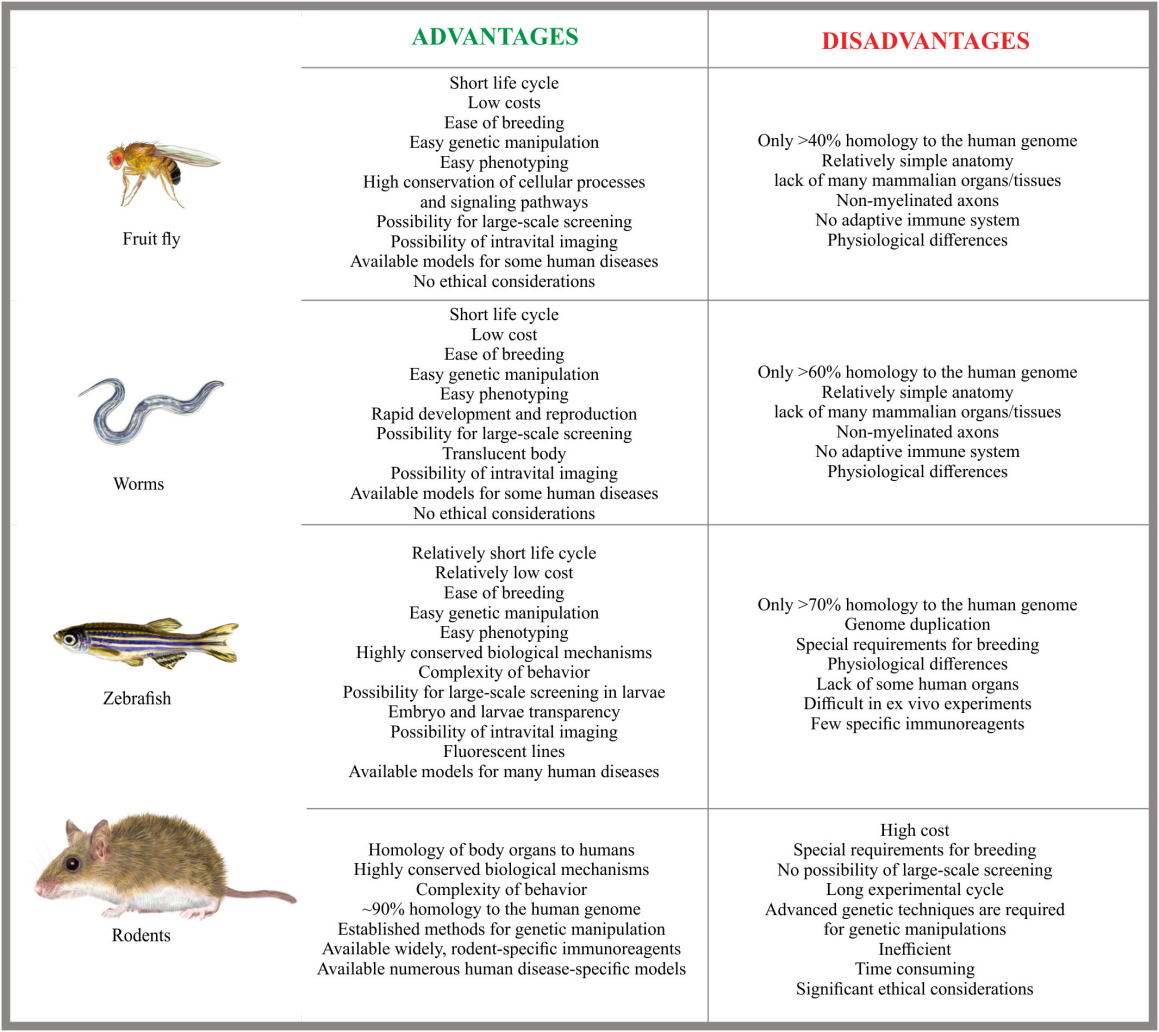
Comparative advantages and disadvantages of animal models in the study of human diseases. This figure presents a side-by-side comparison of commonly used animal models fruit fly, worm, zebrafish, and rodent, in biomedical research. The comparison helps illustrate the strengths and tradeoffs associated with each organism in the context of disease modeling.

CMT are complex diseases that require a multidisciplinary diagnostic and therapeutic approach. Ongoing research and close collaboration among geneticists, neurologists, and other healthcare professionals are essential for advancing the understanding and treatment of these challenging neuropathies. Various model organisms are used in CMT research, each offering distinct advantages. Among *D. melanogaster*, *C. elegans*, and mouse, zebrafish stand out as a particularly valuable laboratory animal due to their unique advantages (Figure 2).

Zebrafish embryos provide a cost-effective and scalable platform for early-stage drug discovery and preclinical testing. Zebrafish can be employed to evaluate compounds or therapies that target or mitigate the effects of genetic variants. Importantly, the zebrafish model not only enables the exploration of genes already associated with CMT but also serves as a crucial tool for investigating the effects of novel or rare genetic variants. Because zebrafish muscles, nerves, visual system, auditory system, cardiac structures, and skeletal components develop rapidly and become functional within 120 hpf, they are particularly well-suited for the rapid assessment of motor deficits, as well as visual, auditory, cardiac, and skeletal abnormalities. In contrast to time- and cost-effective experiments conducted on zebrafish larvae up to 5 dpf, studies of late-onset forms of CMT in adult zebrafish are more demanding but remain valuable. Adult models enable the assessment of disease progression and delayed responses to genetic or pharmacological interventions, thereby significantly advancing our understanding of CMT pathophysiology and therapeutic development. However, their advantage over mammalian models at this stage becomes limited.

It should be noted that gene duplication and the high rate of polymorphism, both common in zebrafish, can complicate genetic analyses. Furthermore, inconsistent nomenclature of some ohnologs and their orthologs continues to cause confusion in comparative genetics and disease modeling (Gasanov et al., 2021). Despite these challenges, the continued application of zebrafish models is expected to substantially contribute to the development of novel therapeutic strategies for disorders within the CMT disease spectrum. A variety of tools – including transgenic lines, antibodies, and dyes are already available for studying CMT; examples are listed in Table 2. Additional resources can be found in an expertly curated and cross-referenced zebrafish research database of the Zebrafish Information Network (ZFIN)^1^.

**TABLE 2:** The list of transgenic lines and antibodies, and dyes used to study Charcot–Marie–Tooth in the zebrafish model.

Transgenic lines	What is labeled	CMT-related work
*Tg(elavl3:EGFP)*	GFP in differentiated neurons (motor and sensory)	Aizawa et al., 2005
*Tg(elavl3:Kaede)*	Kaede in differentiated neurons (motor and sensory)	Sato et al., 2006
*Tg(hb9:eGFP)*	GFP in motoneurons	Chen et al., 2022
*Tg(hb9-MTS-Kaede)*	Photoconvertible Kaede in mitochondria of motor neurons, labeling and ablations	Bergamin et al., 2016
*Tg(isl2b:GFP)*	GFP in retinal ganglion cells	Bragato et al., 2016
*Tg(kdrl:EGFP)s843*	GFP in vasculature	Malissovas et al., 2016
*Tg(mbp:egfp)*	EGFP expressed in mature oligodendrocytes in the embryonic and adult CNS	Jung et al., 2010
*Tg(mbp:gal4-vp16)*	For ablation of oligodendrocytes	Chung et al., 2013
*Tg(mnx1:mCherry)*	mCherry in motor neurons	Mullen et al., 2021
*Tg(ngn1:GFP)*	GFP in sensory neurons	Mullen et al., 2021
*Tg(sensory:GFP)*	GPF in sensory neurites	Eijkenboom et al., 2019b
*Tg(so* × *10:egfp)*	EGFP in oligodendrocyte lineage cells, including OPCs and mature oligodendrocytes	Carney et al., 2006
*Tg(so* × *10:gal4-vp16)*	Used for ablation of oligodendrocytes	Chung et al., 2013
*Tg(uas: nfsB-mCherry)*	Used for the induction of tissue-specific cell death using a bacterial nitroreductase gene under UAS control	Davison et al., 2007
*Tg(uas:egfp)*	Gene trap and enhancer trap	Asakawa et al., 2008
**Dyes**		
Acridine orange	Stains apoptotic cells, 5 μg/mL	Lindzon et al., 2025
α-bungarotoxin (αBTX)	Neuromuscular junction staining, Alexa 488conjugated α-BTX 1:100, Molecular Probes; 1:150 Invitrogen	Lindzon et al., 2025; Ramesh et al., 2010
Rhodamine Phalloidin	F-actin in fast muscles 1:500, Invitrogen	Lindzon et al., 2025; Malissovas et al., 2016
**Antibodies**		
Anti-acetylated tubulin	Mature axons, 1:200, Sigma	Lindzon et al., 2025
Anti-Vinculin	Myotendinous junctions, 1:400, Sigma	Malissovas et al., 2016
Anti-α-Actinin	Sarcomeric z-disks, 1:500	Malissovas et al., 2016
Anti-GARS	Endogenous Gars, 1:3000, Abcam	Malissovas et al., 2016
Anti-p-Eif2a	Phosphorylated Eif2a, 1:250, Cell Signaling Technology	Malissovas et al., 2016
Total-Eif2α	Endogenous Eif2a, 1:500, Cell Signaling Technology	Malissovas et al., 2016
Anti-myosin	Myosin filaments, 1:10, DSHB	Lindzon et al., 2025
Anti-synaptotagmin 2	Primary motor neurons, Znp-1,1:10, DSHB	Lindzon et al., 2025
SV2	Neuromuscular junction staining, 1:50; DSHB	Ramesh et al., 2010

## Footnotes

1
https://zfin.org/action/fish/search
